# Effects of Drying Methods on the Physicochemical and Functional Properties of *Cinnamomum camphora* Seed Kernel Protein Isolate

**DOI:** 10.3390/foods13060968

**Published:** 2024-03-21

**Authors:** Mengqiang Ye, Zhixin Wang, Xianghui Yan, Zheling Zeng, Ting Peng, Jiaheng Xia, Junxin Zhao, Weiyun Wang, Deming Gong, Ping Yu

**Affiliations:** 1 School of Chemistry and Chemical Engineering, Nanchang University, Nanchang 330031, China; 2 State Key Laboratory of Food Science and Resources, Nanchang University, Nanchang 330047, Chinancu_pt@163.com (T.P.);; 3 School of Food Science and Technology, Nanchang University, Nanchang 330031, China; 4 New Zealand Institute of Natural Medicine Research, 8 Ha Crescent, Auckland 2104, New Zealand

**Keywords:** *Cinnamomum camphora* seed kernel protein, drying technique, functional property, physicochemical property

## Abstract

*Cinnamomum camphora* seed kernel protein isolate (CPI) has attracted increasing attention due to its sustainability and potential applications. This study aimed to investigate the effects of freeze-drying (FD), vacuum-drying (VD), and spray-drying (SD) on the physicochemical and functional properties of CPI. The morphology observation results showed that the SD-CPI, SD-CPI, and VD-CPI were spherical, lamellar, and massive, respectively. Compared to FD and SD, VD had more impact on the color, surface hydrophobicity, intermolecular disulfide bonds, intrinsic fluorescence, and thermal stability of CPI. Fourier transform infrared spectroscopy (FTIR) analyses showed that among three CPI samples, VD-CPI had the highest content of β-sheet but the lowest contents of α-helix and β-turn. At different pH values, the solubility, emulsification, and foaming properties of VD-CPI were inferior to those of FD-CPI and SD-CPI. These results provide useful information on the changes in the physicochemical and functional properties of CPI subjected to different drying methods, and offer theoretical guidance for the production and use of CPI in the food industry.

## 1. Introduction

*Cinnamomum camphora*, commonly known as the camphor tree, belongs to the family *Lauraceae* and is a large evergreen broadleaf tree found in subtropical and tropical regions of Asia. In southern China, it is extensively planted as a landscaping tree, with an annual seed yield exceeding 1 million tons [1]. *Cinnamomum camphora* seed kernel (CCSK) is a significant source of medium-chain oils and proteins, constituting approximately 59% and 19%, respectively. Additionally, CCSK serves as a rich source of fibers, minerals, and phytochemical constituents, such as polyphenols and flavonoids [2]. It has been reported that the *Cinnamomum camphora* seed kernel protein isolate (CPI) contained a high level of essential amino acids (39.25%), with notable concentrations of leucine (56.5 g/kg) and tyrosine (47.9 g/kg), rendering it nutritionally comparable to soy protein [1]. The essential amino acid profile of CPI complies with the recommended values for adults set by the FAO and WHO, positioning it as a natural and high-quality plant protein supplement. In addition to its high nutritional value, CPI also exhibited good emulsification and foaming abilities, as well as high solubility [1].

The variations in the structural and compositional heterogeneity of proteins from different plant sources result in differences in their functionalities, such as solubility, gelation, water/oil absorption capacity, and the stability of emulsions and foams [3]. The application of plant proteins in food processing largely depends on their functional properties, which, in turn, are influenced by the protein extraction process and drying techniques. Drying serves the purpose of enhancing the storage stability of proteins, ensuring that the nutritional components of proteins remain intact. Freeze-drying (FD), vacuum-drying (VD), and spray-drying (SD) are typical and widely used drying techniques in the processing of food proteins [4]. However, these drying methods involve completely different drying conditions (e.g., temperature, pressure, and time), which may have a significant influence on the chemical composition and functional properties of proteins. FD involves the removal of moisture in a frozen substance to turn directly into gas at a low pressure, which can maintain the original quality in a substance but requires a significant amount of energy. VD is achieved by removing moisture from a substance under relative high temperature and vacuum conditions, which may lead to the loss of heat-sensitive components in a substance. SD involves atomizing liquids into small particles and drying rapidly in a hot air stream, which is efficient for large-scale production but may result in a loss of heat sensitivity. It has been reported that different drying methods significantly impact the final functional properties of plant proteins [5]. For instance, Hu et al. (2009) [6] found that soy protein isolate produced via SD exhibited lower denaturation compared to FD and VD, with superior solubility and excellent emulsifying and foaming properties. Similarly, Joshi et al. (2011) [7] reported that compared to VD and FD, the lentil protein isolate obtained via SD had a higher solubility and gelling properties but a lower water uptake capacity. Zhao et al. (2013) [8] observed that a rice protein isolate obtained through SD had a higher protein solubility, emulsifying activity, and foaming capacity compared to FD. On the other hand, Shen et al. (2021) [4] found that compared to the SD and VD processes, a quinoa protein isolate formed via the FD process underwent less denaturation and exhibited better functional properties. These studies suggest that the functional properties of proteins may be affected not only by drying methods, but also by the structure of the protein itself. To the best of our knowledge, little is known about the effects of different drying methods on the structure and functionality of CPI.

This study aimed to investigate the effects of different drying methods (FD, VD, and SD) on the amino acid composition, protein structures, functional properties, and volatile components of CPI. The physicochemical properties were characterized by determining the microscopic morphology, particle size, surface hydrophobicity, SDS/Native-PAGE, intrinsic fluorescence spectra, and Fourier transform infrared spectroscopy (FTIR). Functional properties, including the solubility, emulsifying capacity, and foaming capacity, were measured. The selection of an appropriate drying method will facilitate the industrial optimization of protein production and the targeted application of functional properties, promoting the effective utilization of CPI in the food industry.

## 2. Materials and Methods

### 2.1. Materials

The *Cinnamomum camphora* seeds used in this study were collected from the Qingshan Lake Campus of Nanchang University, Nanchang, China. Sodium dodecyl sulfate (SDS) and ethylenediaminetetraacetic acid (EDTA) were purchased from Shanghai Aladdin Reagent Co., Ltd. All gel electrophoresis reagents were from Beijing Solarbio Technology Co., Ltd. Other reagents were analytical grades.

### 2.2. Separation of the Cinnamomum camphora Seed Kernel Protein Isolate (CPI)

*Cinnamomum camphora* seeds were peeled, dried, and shelled to obtain the *Cinnamomum camphora* seed kernel (CCSK). Then, CPI was prepared from CCSK according to our previous study [1]. Briefly, CCSK was crushed and degreased with n-hexane in a ratio of 1:3 (*w*/*v*) at room temperature. The mixture was stirred continuously for 12 h, this was repeated twice, and then the mixture was separated with a suction filter pump. The filtrate was discarded, and the residues were air-dried at room temperature in a fume hood to obtain degreased CCSK. The degreased CCSK was dispersed in 80% (*v*/*v*) ethanol with a powder/liquid ratio of 1:20 (*w*/*v*), stirred at room temperature for 2 h, and then centrifuged at 3500× *g* for 15 min. The precipitate was resuspended in the solvent, and the extraction was repeated three times. Then, the defatted and dephenolized CCSK meal was used to extract CPI by using the methods of alkali extraction (pH 9.0) and acid precipitation (pH 5.0). After the precipitate was resuspended in distilled water and the pH value readjusted to 7.0, followed by centrifugation at 3500× *g* for 15 min, the CPI solution was obtained for drying treatments.

### 2.3. Drying Treatments

Freeze drying CPI (FD-CPI): The CPI solution was frozen at −80 °C for 12 h, then the frozen sample was lyophilized at −80 °C by using a freeze dryer (SCIENTZ-18N, Scientz Biotechnology Co., Ltd., Ningbo, China) for 48 h.

Vacuum drying CPI (VD-CPI): The CPI solution was dried directly in the vacuum oven (DZF-6050, CIMO Medical Instrument Manufacturing Co., Ltd., Shanghai, China) at 0.09 MPa and 60 °C for 48 h.

Spray drying CPI (SD-CPI): The CPI solution was loaded on a laboratory-scale centrifugal spray dryer (B-290, BUCHI Co., Ltd., Shanghai, China) with an inlet temperature of 120 ± 5 °C, an outlet temperature of 70 ± 5 °C, and an injection flow rate of 0.5 L/h.

The three dried CPI samples were ground, sifted, and kept at −20 °C for further analysis. The protein content of CPI samples was determined via an automatic Kjeldahl nitrogen analyzer (K9860, Haineng Future Technology Group Co., Ltd., Jinan, China) with a conversion coefficient of 6.25.

### 2.4. Determination of Physicochemical Properties of CPI

#### 2.4.1. Color Measurement

The color of CPI samples was determined by using a colorimeter (LS171, Linshang Technology Co., Ltd., Shenzhen, China) with a white tile [L* (lightness) = 94.55, a* (green) = −0.24, b* (yellow) = 2.16] as a reference. The results were expressed as the color parameters of L*, a*, b* and **Δ***E*.

#### 2.4.2. Scanning Electron Microscopy (SEM)

A scanning electron microscope (SU8020, Hitachi, Ltd., Tokyo, Japan) was used to observe the surface morphology of CPI samples. The samples were sprayed with a conductive layer of gold under vacuum conditions, the SEM images were obtained with an accelerating voltage of 3 kV, and the magnifications were set as 200, 1000, and 5000 times.

#### 2.4.3. Measurement of Free Sulfhydryl (-SH) Group Content

The -SH contents of CPI samples were determined according to Ellman’s DTNB method [9]. Briefly, 75 mg of each protein sample was completely dissolved in 5.0 mL of Tris-Gly buffer containing 8 M urea (pH 8.0, 0.086 M Tris, 0.09 M Gly, and 0.004 M EDTA). Then, 50 μL Ellman reagent (4.0 mg DTNB, 1.0 mL Tris-Gly) was added, and the mixture was incubated at room temperature for 1 h. After centrifugation at 8000× *g* for 15 min, the supernatant was collected and the absorbance was measured at 412 nm. The -SH content of each sample was calculated as follows:

-SH (μmol/g) = (73.53 × D × A_412_)/C (1)

where 73.53 is a coefficient derived from 10^6^/(1.36 × 10^4^); 1.36 × 10^4^ and 10^6^ are the conversions from the molar basis to the μmol/mL basis and from mg protein to g protein, respectively; D is the dilution factor; and A_412_ is the absorbance at 412 nm.

#### 2.4.4. Determination of Particle Size

The particle sizes of CPI samples were measured using the Zetasizer nanosystem (Malvern Instruments, Worcestershire, UK) at room temperature, according to our previous study [1]. Briefly, the protein sample was prepared in a 1 mg/mL solution with ultrapure water, and possible impurities were removed via filtration through a 0.45 μm polyethersulphone membrane. The refractive indices of the protein and water were set to 1.46 and 1.33, respectively. All measurements were repeated three times.

#### 2.4.5. Determination of Surface Hydrophobicity (H_0_)

The H_0_ was determined according to the method of Yan, Gong et al. (2022) [10], with slight modification. Briefly, the protein sample was dissolved in ultrapure water at 0.05–0.25 mg/mL. Then, 1 µL of 8 mmol/L ANS solution was mixed with 200 µL sample solution in a 96-well plate. Fluorescence intensity was recorded using a microplate reader (Varioskan LUX, ThermoFisher Scientific, Waltham, MA, USA) with an excitation wavelength of 390 nm and an emission wavelength of 470 nm. H0 represents the linear regression slope of fluorescence intensity versus protein concentration.

#### 2.4.6. FTIR

The FT-IR spectra of CPI samples were measured via a Nicolet iS50 spectrometer (Thermo Nicolet Co., Waltham, MA, USA) according to a previous study [11]. Lyophilized samples were mixed with KBr in a ratio of 1:100 (*w*/*w*) and pressed. The spectra of samples were recorded by scanning between 400 and 4000 cm^− 1^ at room temperature. The resolution of each spectrum was 4 cm^− 1^ with 64 scans.

#### 2.4.7. Intrinsic Fluorescence Spectrum Measurement

The intrinsic fluorescence of CPI samples was determined via the method of Yan and Gao et al. (2021) [12], with slight modification. Briefly, 1 mg/mL of sample solution was prepared by dissolving protein sample in ultra-pure water. After removing the impurities via filtration with 0.45 µm polytetrafluoroethylene membrane, the fluorescence spectrum was determined by using a fluorescence spectrophotometer (F-7000, Hitachi High-Tech Co., Ltd., Shanghai, China) with excitation wavelength of 280 nm, emission wavelengths of 290–500 nm, and slit width of 5 nm.

#### 2.4.8. SDS-PAGE and Native-PAGE

SDS-PAGE and Native-PAGE were performed according to the method of Yan and Gao et al. (2021) [12], with minor modifications. For SDS-PAGE, an electrophoretic system with 5% stacking gel and 12% resolving acrylamide gel was used. Samples (2 mg/mL) were prepared under reducing (with β-mercaptoethanol) and non-reducing (without β-mercaptoethanol) conditions, respectively. Then, 10 μL of sample and protein standards (10–180 kDa) were added to the corresponding loading wells, respectively. The electrophoresis was firstly performed at 80 V constant pressure toward the resolving gel and then at 120 V constant pressure toward the bottom.

For Native-PAGE, electrophoresis system with 5% stacking gel and 12% resolving acrylamide gel without SDS was used. An aliquot of 40 μL protein solution (2 mg/mL) was mixed with 10 μL sample buffer containing 65.8 mM Tris-HCl (pH 6.8), 26.3% (*w*/*v*) glycerol, and 0.01% bromophenol blue. Then, 10 µL of sample and protein standards (11–245 kDa) were added to the appropriate gel pores, respectively. Electrophoresis was firstly performed at 100 V constant pressure toward the resolving gel and then at 160 V constant pressure toward the bottom.

After electrophoresis, the gels were stained with Coomassie Blue R250 and then destained with a destaining solution (distilled water: ethanol: acetic acid = 7:2:1, *v*/*v*/*v*). A ChemiDoc Touch Imaging System (Bio-Rad Laboratories, Hercules, CA, USA) was used for scanning, and Image Lab software (Version 6.1) was used for analysis.

#### 2.4.9. X-ray Diffraction (XRD) Measurement

The XRD patterns of CPI samples were measured by using a PANalytical Empyrean Series 2 diffractometer (Malvern Panalytical, Malvern City, UK) with Cu Kα radiation at a ratio of 0.5. The samples were removed from the vials and flattened onto a glass slide, which was then scanned from 5° to 90° (2θ) at a rate of 2°/min at room temperature.

#### 2.4.10. Thermal Behavior

Differential scanning calorimetry (DSC): CPI samples were measured via DSC250 (TA Instruments, New Castle, DE, USA) according to the method described by Yan and Gong et al. (2022) [10], with minor modifications. Briefly, 5–7 mg of sample was weighed and placed in an aluminum crucible, and an empty aluminum crucible was used as control. DSC analysis was performed from 25 to 200 °C at a heating rate of 10 °C/min. The data were analyzed by using TRIOS software (Version 5.0).

Thermogravimetric analysis (TGA): TGA analysis of CPI samples was performed by using the STA 2500 Regulus analyzer (NETZSCH Group, Bavarian, Germany). Protein samples were heated in an aluminum pot from 30 °C to 600 °C at a heating rate of 10 °C/min [13].

### 2.5. Functional Properties of CPI

#### 2.5.1. Solubility

The protein solubility of CPI samples was measured according to Hadnađev et al. (2018) [14], with some modifications. Briefly, each protein sample was dissolved in distilled water to 1 mg/mL, then the pH value of the dispersion was adjusted to 3–12 with 1.0 M HCl or 1.0 M NaOH. The dispersions were then centrifuged at 10,000× *g* for 10 min, and the protein content of the supernatant was determined via the Bradford assay.

#### 2.5.2. Emulsifying Properties

The emulsifying activity index (EAI) and emulsifying stability index (ESI) of CPI samples were determined according to Zhong et al. (2012) [15], with slight modifications. Briefly, protein samples were dissolved in distilled water to produce protein solutions at 2 mg/mL. The pH value of the solution was adjusted to 3–12 using either 1.0 M HCl or 1.0 M NaOH. Aliquots of 15 mL of protein solution were individually mixed with 5 mL of soybean oil. The mixture was homogenized at a speed of 13,600 rpm at room temperature for 2 min by using a T18 digital ULTRA-TURRAX (IKA Instrument Equipment Co., Ltd., Guangzhou, China). Subsequently, 50 μL was taken from the bottom of the homogenized emulsion and dispersed in a 5 mL of 0.1% sodium dodecyl sulfate (SDS) solution at 0 min and 10 min, respectively. The absorbance of mixtures was measured at 500 nm by using a UV–visible spectrophotometer (UV-1950, Puxi General Instrument Co., Ltd., Beijing, China). A 0.1% (*w*/*v*) SDS solution was used as a blank control. The emulsifying activity index (EAI) and emulsion stability index (ESI) were calculated by using the following formulas:

EAI (m^2^/g) = (2 × 2.303 × A_0_ × DF)/(c × φ × 10,000) (2)

ESI (%) = (A_10_/A_0_) × 100 (3)

where A_0_ and A_10_ represent the absorbance at 500 nm measured at 0 and 10 min for the emulsion, respectively. DF is the dilution factor (101), C is the protein concentration before emulsification (g/mL), and φ is the volume fraction of oil in the emulsion (*v*/*v*) (φ = 0.25).

#### 2.5.3. Foaming Capacity and Stability

The foaming properties of CPI samples were determined according to a previous method [16], with some modifications. Briefly, the protein samples were dissolved in distilled water to produce protein solutions at 10 mg/mL. The pH value of the solution was adjusted to 3–12 with 1.0 M HCl or 1.0 M NaOH and then homogenized at a speed of 13,600 rpm at room temperature for 2 min by using the T18 digital Ultra-TURRAX (IKA, Instrument Equipment Co., Ltd., Guangzhou, China). The foam volume was measured at 0 min (V_0_) and 10 min (V_10_) after homogenization. The foam capacity (FC) and foam stability (FS) were calculated by using the following formulas:

FC (%) = (V_0_ − V)/V × 100 (4)

FS (%) = (V_10_/V_0_) × 100 (5)

### 2.6. Statistical Analysis

Results are expressed as mean ± standard deviation. Data were analyzed by one-way analysis of variance (ANOVA), followed by Tukey’s test using SPSS 26.0 software for post hoc analysis. The significance of differences was defined at *p* < 0.05.

## 3. Results and Discussion

### 3.1. Physicochemical Properties of CPI

#### 3.1.1. Protein Contents and Colors of CPI

**Figure 1:**
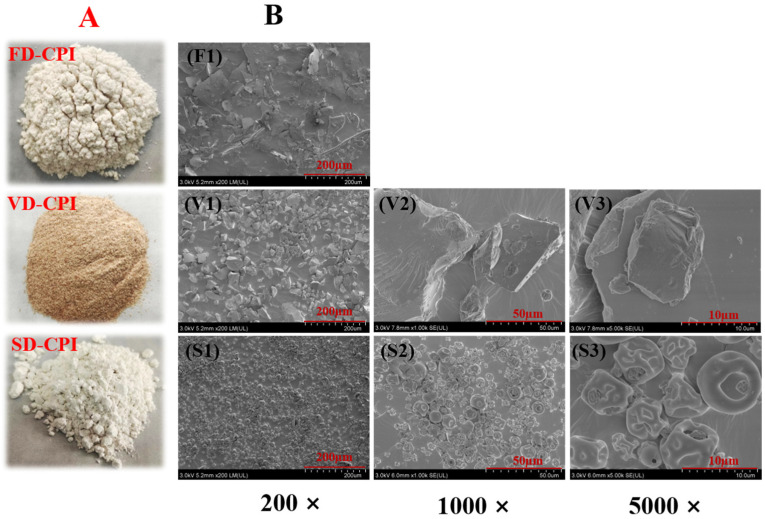
The appearances (**A**) and SEM images (**B**) of FD-CPI, VD-CPI, and SD-CPI. F1 indicates SEM image of FD-CPI at magnification of 200×. V1–V3 and S1–S3 indicate SEM images of VD-CPI, and SD-CPI at different magnifications (200×, 1000×, and 5000×), respectively.

As shown in Table 1, the protein content of VD-CPI (87.52 ± 0.70%) was significantly lower (*p* < 0.05) than those of FD-CPI (93.24 ± 0.30%) and SD-CPI (92.18 ± 1.46%). Notably, the protein contents of FD-CPI and SD-CPI were 5.72% and 4.66% higher than that of VD-CPI, respectively. This difference may be attributed to the higher moisture content in VD-CPI compared to the other samples, as evidenced by the results of TGA. There were observable differences in the external color of the three protein samples (Figure 1A). In detail, SD-CPI had the highest L* value (55.53 ± 0.30) and the lowest a* value (0.57 ± 0.12) and b* value (0.25 ± 0.06). In contrast, VD-CPI had the lowest L* value (35.81 ± 0.10) and the highest a* value (1.65 ± 0.18) and b* value (4.05 ± 0.09). The L* value (40.25 ± 0.13), a* value (1.56 ± 0.49), and b* value (2.55 ± 0.06) of FD-CPI were between those of SD-CPI and VD-CPI. The color of protein powders can be collectively influenced by particle size distribution, intrinsic properties, purity, pigment content, and the removal of pigments during processing [4]. SD-CPI showed the highest whiteness and brightness, possibly due to the brighter color resulting from the presence of heat-labile pigments during the spray-drying process, resulting in a lower browning index [17] and the smallest particle size [6]. VD-CPI showed less whiteness and a darker color, possibly due to the formation of Maillard reaction products resulting from the interaction of amino groups with carbonyl compounds during the prolonged heating [3,18]. Moreover, the Δ*E* value of VD-CPI was the highest (58.80 ± 0.11), followed by FD-CPI (54.33 ± 0.11) and SD-CPI (39.07 ± 0.48), indicating that different drying methods had a great influence on the color of the sample. The results were also consistent with the visual appearance of the sample (Figure 1A).

#### 3.1.2. SEM

As shown in Figure 1B, there were significant differences in the surface morphologies of FD-CPI, VD-CPI, and SP-CPI. Specifically, FD-CPI had a large and thin layered structure (F1, 200×), which is the typical morphology of most lyophilized plant proteins, possibly due to the formation of solute aggregates caused by intermolecular forces, such as covalent bonds, electrostatic interactions, and hydrophobic interactions during drying [19,20]. Moreover, the ice sublimation stage of FD has a significant effect on the shape and volume of the powder due to the formation of large pores [21,22]. However, the morphology of VD-CPI comprised randomly crushed stone particles (V1, 200×), and that of SD-CPI comprised finely crumpled spheroid particles (S1, 200×). VD-CPI showed a thick and dense lumpy structure (V2, 1000×), which may be caused by the layering of solutes and solvents during a long heating and drying process, with proteins gradually settling to the bottom of the container while water was transferred from the interior of the sample to the surface and rapidly evaporated [23]. The microstructure of SD-CPI was spherical and pleated (S3, 5000×), which may be related to the characteristics of spray-drying. The protein solution entered the drying chamber in the form of small droplets and came into direct and rapid contact with hot and dry air. The instantaneous drying temperature was high, and the small droplets rapidly lost water to form spherical particles. The wrinkles in SD-CPI may be attributed to the rapid evaporation of water and the formation of a protective film on the droplet surface during the spray-drying process, resulting in uneven shrinkage [19,24].

#### 3.1.3. -SH Content, Particle Size, and H_0_

Free sulfhydryl (-SH) plays a significant role in protein conformation and impacts the functional performance of proteins [25]. The -SH group content of VD-CPI (4.86 ± 0.03 µmol/mL) was significantly lower (*p* < 0.05) than those of FD-CPI (5.32 ± 0.01 µmol/mL) and SD-CPI (5.24 ± 0.11 µmol/mL) (Table 2). This discrepancy may be attributed to the formation of intermolecular disulfide bonds of CPI under VD conditions, including relatively a higher temperature and longer time, resulting in the formation of protein aggregates [26]. This phenomenon was in agreement with the results of SDS-PAGE profiles. Additionally, the SD process involves a higher drying temperature but a shorter drying time, which may prevent the formation of intermolecular disulfide bonds. Therefore, SD-CPI showed similar -SH group content to FD-CPI.

The degree of aggregation and conformational changes in proteins can be inferred from their particle size [27]. As shown in Table 2, the average particle size of SD-CPI (84.70 ± 1.22 nm) was significantly smaller (*p* < 0.05) than those of FD-CPI (117.70 ± 1.66 nm) and VD-CPI (122.10 ± 4.33 nm). On the other hand, the average particle sizes of FD-CPI and VD-CPI were 0.39 and 0.44 times higher than (*p* < 0.05) that of SD-CPI, respectively. This may be because the SD process involves a high temperature but short duration, thereby resulting in minimal protein denaturation and limited protein polymerization. VD-CPI exhibited the largest average particle size, which may be because the long-term heat treatment accelerated the movement of protein molecules and thereby increased the probability of protein collision, ultimately resulting in an increase in protein aggregates and the average particle size [28]. This observation was consistent with the result of SEM (Figure 1B).

H_0_ is an indicator of the degree of exposed hydrophobic amino acid residues on the protein surface, which also determines the functional properties of proteins [29]. As shown in Table 3, the H_0_ values of the three CPI samples varied significantly (*p* < 0.05); FD-CPI had the highest H_0_ value (85.99 ± 6.79), followed by SD-CPI (70.60 ± 7.10) and VD-CPI (52.59 ± 0.01). Similarly, a study by Shen et al. (2021) [4] on quinoa protein isolate found that the H_0_ value of the FD protein was the highest, while that of the VD protein was the lowest. This may be because the FD protein had undergone some degree of denaturation, resulting in the exposure of hydrophobic groups. On the other hand, the VD protein showed more intense denaturation due to hydrophobic exchange reactions between the protein molecules. This phenomenon may be associated with the formation of a film on the protein surface, leading to protein aggregation and low surface hydrophobicity [4].

#### 3.1.4. FTIR

**Figure 2:**
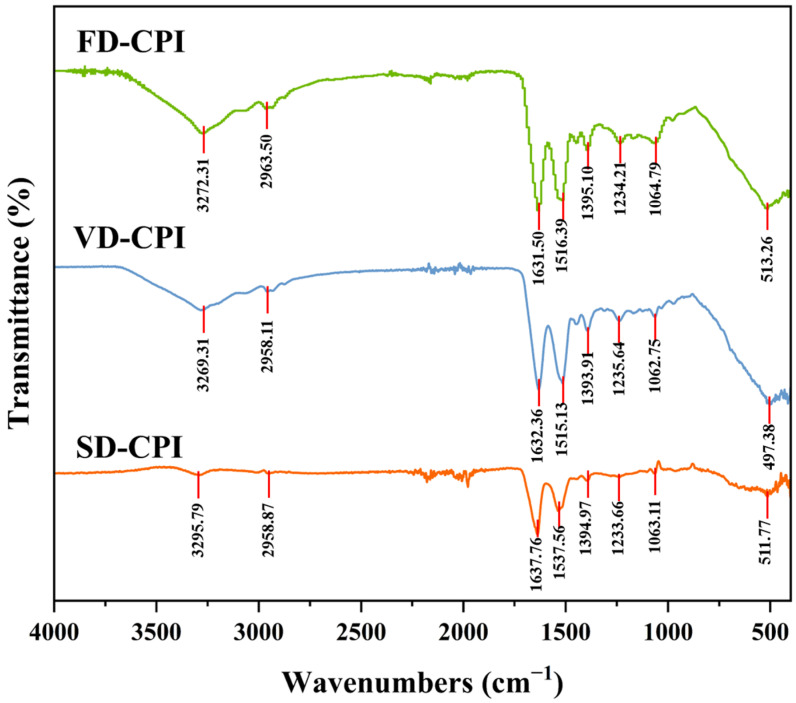
FTIR spectra of FD-CPI, VD-CPI, and SD-CPI.

Fourier transform infrared spectroscopy is a common tool for predicting the secondary structure of proteins [18]. Compared to VD-CPI and FD-CPI, SD-CPI showed significant differences in the peak positions and intensities of infrared spectra (Figure 2). Specifically, the peak positions of the amide A (3450–3300 cm^−1^), amide I (1700–1600 cm^−1^), and amide II (1600–1500 cm^−1^) bands of SD-CPI showed a significant red shift, accompanied by a reduction in peak intensities. The amide A band is a typical stretching vibration of N-H hydrogen bonding. Hence, the decrease in peak intensity at the amide A band of SD-CPI may be due to the weakening of hydrogen bonds caused by elevated temperatures [18]. High temperatures may induce conformational changes within protein molecules, leading to the breakage and formation of hydrogen bonds and, consequently, affecting the vibrational frequencies of the amide A band.

The secondary structures of proteins in the amide I band were observed at 1615–1637 cm^−1^ and 1682–1700 cm^−1^ (β-sheet), 1637–1648 cm^−1^ (random coil), 1648–1664 cm^−1^ (α-helix), and 1664–1682 cm^−1^ (β-turn) [30]. As shown in Table 3, the secondary structure of three CPI samples was found to be primarily comprised of β-sheet (33.33–42.38%), followed by α-helix (22.65–27.85%) and random coil (15.38–21.01%). Similarly, previous researches on plant proteins, such as black bean protein [31], pea protein [31,32], and hempseed protein [33], have also reported that the β-sheet was the dominant secondary structure, followed by α-helix or random coil. Notably, compared to FD-CPI (42.38%) and VD-CPI (44.47%), SD-CPI had a significant lower (*p* < 0.05) β-sheet content (33.33%). This is because under high-temperature conditions, the occurrence of hydrogen bonding and hydrophobic interactions may have led to the disruption or degradation of the β-sheet structure [34]. Similarly, Dong et al. (2024) [35] reported that the β-sheet content of a hempseed protein isolate subjected to SD process was significantly lower than that subjected to FD process. However, a conflicting result was observed by Shen et al. (2021) [4] on a quinoa protein isolate. The conflicting result emphasized that the effect of drying methods on protein secondary structures may be also related to the protein source. In addition, FD-CPI showed a lower amount of random coil compared to VD-CPI and SD-CPI. This phenomenon can be attributed to the relative higher drying temperatures in the VD and SD process, thus leading to the appearance of additional random coils [36].

#### 3.1.5. SDS-PAGE and Native-PAGE Analysis

**Figure 3:**
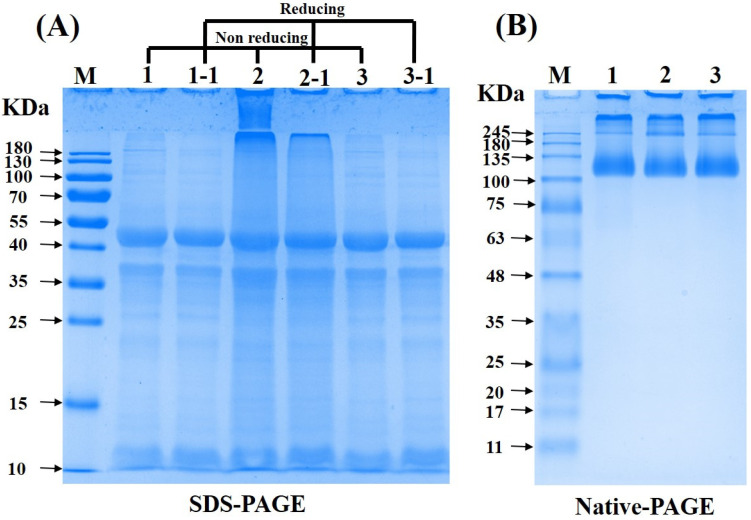
SDS-PAGE profile under non-reducing conditions (**A**, lanes 1, 2, and 3), SDS-PAGE profile under reducing conditions (**A**, lanes 1-1, 2-1, and 3-1) and native-PAGE (**B**, lanes 1, 2, and 3) of FD-CPI, VD-CPI, and SD-CPI. Lanes 1 (1-1), 2 (2-1), and 3 (3-1) represent the samples of FD-CPI, VD-CPI, and SD-CPI, respectively. M represents the protein markers (10–180 kDa).

As shown in Figure 3A, under reducing conditions, the three CPI samples exhibited a great similarity in their electrophoresis profiles that included several major bands: 40–55 kDa, 35–40 kDa, and 10 kDa. This result was in accordance with our previous studies [12,30]. However, it was observed that VD-CPI differed from the other samples by the presence of additional protein bands exceeding 180 kDa. Consistently, under non-reducing conditions, only VD-CPI exhibited protein bands with a higher molecular weight (more than 180 kDa). These results suggested that a partial aggregation occurred in VD-CPI to form high-molecular-weight macromolecules through disulfide bonds, which was also evidenced by the result for the -SH group contents (Table 3). Accordingly, the VD process takes a longer time at a relatively higher temperature, which may lead to severe protein denaturation, aggregation, and crosslinking [4].

The result of native-PAGE can indicate both the quantity and structural characteristics of native proteins [37]. As shown in Figure 3B, the electrophoretic profiles of three CPI samples were similar, with distinct concentrated protein bands at approximately 130 kDa and 245 kDa. However, VD-CPI showed a darker band around 245 kDa compared to FD-CPI and SD-CPI. This may be due to the intermolecular cross-linking induced by the VD process, leading to the formation of large molecular polymers. This observation was also consistent with the results for the -SH group contents (Table 3). In summary, based on the results of SDS-PAGE and native-PAGE, only the VD process had significant effects on the molecular weight of CPI.

#### 3.1.6. Intrinsic Fluorescence Spectrum

**Figure 4:**
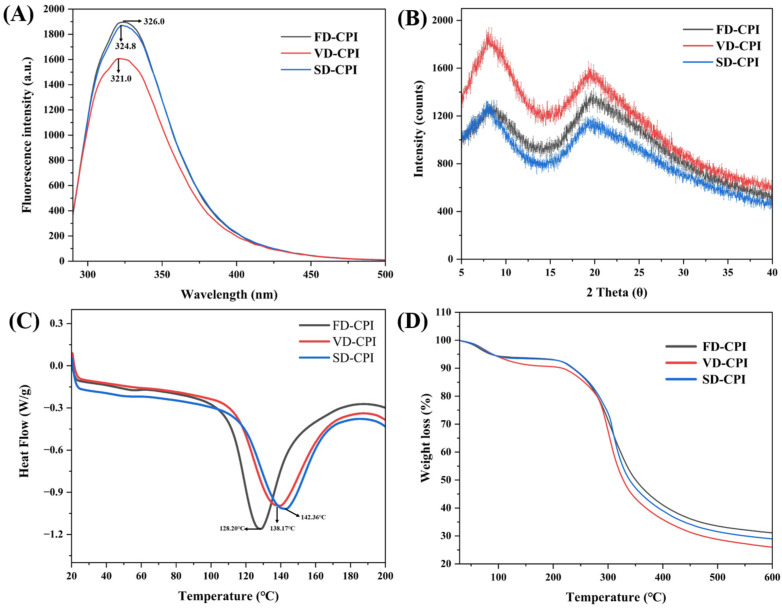
Intrinsic fluorescence spectra (**A**), X-ray diffraction pattern (**B**), differential scanning calorimetry pattern (**C**), and thermogravimetric analysis (**D**) of FD-CPI, VD-CPI, and SD-CPI.

The measurement of intrinsic fluorescence can reflect the changes in the tertiary structure of proteins [38]. This measure is based on the sensitivity of aromatic amino acid residues (especially tryptophan residue) to the polarity of the surrounding microenvironment [39,40]. As shown in Figure 4A, the fluorescence intensities of FD-CPI and SD-CPI appeared similar, whereas VD-CPI showed a significant reduction in fluorescence intensity. This phenomenon may be due to the partially aggregation and cross-linking of CPI subjected to the VD process, as confirmed by the results for the -SH group contents (Table 3) and SDS-PAGE profiles (Figure 3). In a study of the *Rana chensinensis* ovum protein isolate, Li et al. (2022) [23] observed similar fluorescence intensities in FD and SD proteins, whereas the protein subjected to VD showed a significant decrease in fluorescence intensity. In addition, the sequential increase in the maximum emission wavelengths (λ_max_) for VD-CPI (321.0 nm), SD-CPI (324.8 nm), and FD-CPI (326.0 nm) indicated that the tryptophan residues were exposed to a more hydrophilic environment, facilitating their interaction with ANS. This observation was consistent with the results of H_0_ (Table 2).

#### 3.1.7. XRD

X-ray diffraction is used to study changes in the protein crystal structure. As shown in Figure 4B, all CPI samples exhibited two characteristic X-ray diffraction peaks around 2θ = 10° and 2θ = 20°. The intensity of diffraction peaks is related to the crystallinity or ordered arrangement of the protein structure [41]. VD-CPI showed a significantly enhanced diffraction peak intensity at 2θ = 10° compared to FD-CPI and SD-CPI. Similarly, Joshi et al. (2011) [7] found that the diffraction peak intensity of VD lentil protein was higher than that of SD and FD lentil protein. This result may be due to an increase in the crystalline structure of the proteins under VD conditions, leading to a significant increase in the diffraction peak intensity [7].

#### 3.1.8. Thermal Stability

##### DSC Analysis

DSC is frequently utilized to gauge the degree of denaturation, structural changes, and conformational modifications in protein molecules. The DSC curves of three CPI samples are shown in Figure 4C, and the thermal parameters, including denaturation peak temperature (T_d_) and enthalpy (ΔH), are shown in Table 3. T_d_ serves as an indicator of protein thermostability, while ΔH is a measure of the hydrophobic and hydrophilic interactions of protein molecules [42] and correlates with the degree of ordered structure within proteins. The thermograms of all CPI samples showed a single endothermic peak. The T_d_ values of FD-CPI, VD-CPI, and SD-CPI were 128.20, 138.17, and 142.36 °C, respectively, suggesting that CPI obtained via the VD and SD processes had higher thermal stability. In addition, the ΔH value of VD-CPI (117.43 J/g) was higher than those of SD-CPI (101.38 J/g) and FD-CPI (98.79 J/g). This is because during the VD process, the longer drying time may lead to the massive denaturation of protein samples, with many intermolecular bonds breaking and new protein–protein interactions forming denser structural features that require a higher temperature and energy for the protein to denature [36]. Furthermore, the occurrence of the Maillard reaction under VD conditions may contribute to achieving the highest T_d_ [43]. Similarly, proteins isolated from fenugreek (*Trigonella foenum graecum*) [43] and grass pea [44] subjected to VD also exhibited increased thermal stability than those subjected to SD and FD.

##### TGA Analysis

TGA is commonly employed to investigate thermal degradation behavior and confirm the presence of structural changes. As shown in Figure 4D, during the heating process, all protein samples exhibited a pronounced loss of mass. The initial phase of mass loss occurred from room temperature to around 200 °C, attributing to the evaporation of free and bound water. Both FD-CPI and SD-CPI exhibited similar mass reductions at this stage, approximately 7% by weight. However, VD-CPI showed a larger disparity, losing around 10% of its weight, likely due to its higher moisture content compared to the other samples. In the second temperature range (200–600 °C), the maximum mass loss of the samples occurred, attributing to the degradation of protein structures and the release of low-molecular-weight volatile compounds [19].

### 3.2. Functional Properties of CPI

#### 3.2.1. Solubility

**Figure 5:**
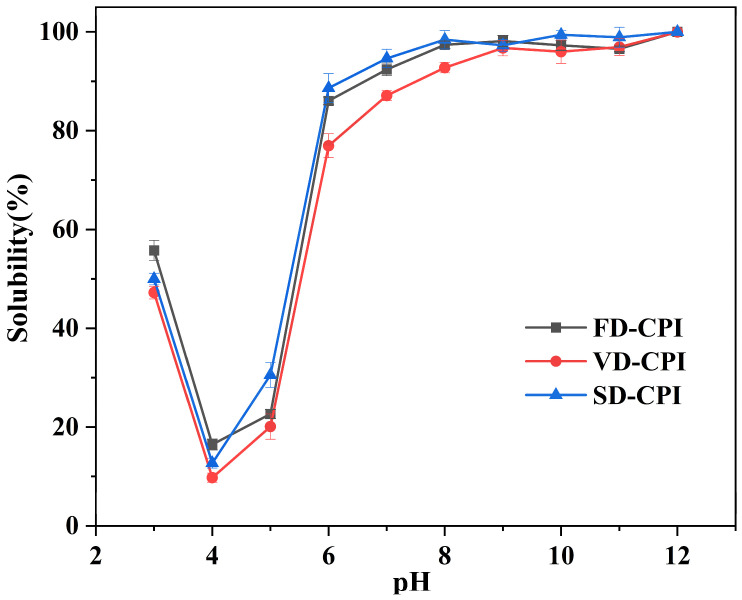
Solubility curves of FD-CPI, VD-CPI, and SD-CPI at pH values of 3–12.

The solubility of proteins is a manifestation and outcome of the strong interactions between polar groups in proteins and water molecules and serves as a prerequisite for many other functional properties of proteins. The solubility curves of the samples from pH 3 to 12 were both U-shaped (Figure 5). Previous studies on FD-CPI showed a similar trend of solubility [12,30]. All CPI samples exhibited minimum solubility at pH 4 (Figure 5), which could be because pH 4 was near the isoelectric point of CPI. In addition, the solubility profiles of FD-CPI and SD-CPI were similar, while VD-CPI exhibited significantly lower (*p* < 0.05) solubility at pH 6–8. Notably, the solubility of CPI was 15.16%, 8.62%, and 6.10% higher after the SD process in relation to the VD process at pH 6, 7, and 8, respectively. This result was consistent with the finding reported by Li et al. (2022) [23], which may be due to the extensive denaturation of CPI caused by the VD process during a long heating period. This denaturation may promote hydrophobic interactions between protein molecules, resulting in the formation of a surface moisture barrier and reducing protein solubility [4].

#### 3.2.2. Emulsifying Capacity and Stability

**Figure 6:**
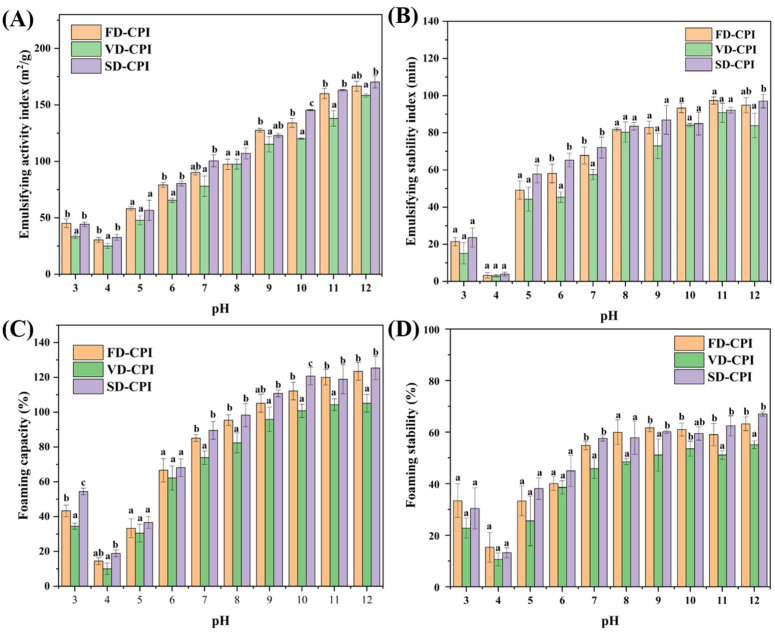
Emulsifying properties (**A**,**B**) and foaming properties (**C**,**D**) of FD-CPI, VD-CPI, and SD-CPI at pH values of 3–12. Means with different superscript letters (a, b, c) within the same pH value are significantly different (*p* < 0.05).

The emulsifying capacity index (EAI) and emulsifying stability index (ESI) of proteins play an indispensable role in the food industry [44]. As shown in Figure 6A,B, the EAI values of FD-CPI, VD-CPI, and SD-CPI at different pH levels ranged from 30.44 to 176.68 m^2^/g, 25.00 to 158.31 m^2^/g, and 32.66 to 163.32 m^2^/g, respectively. The ESI values of FD-CPI, VD-CPI, and SD-CPI ranged from 5.25% to 97.39%, 2.92% to 90.82%, and 1.25% to 92.18%, respectively. Notably, VD-CPI showed significantly lower (*p* < 0.05) EAI values compared to FD-CPI and SD-CPI, which could be attributed to its lower protein solubility, since the trends in EAI and ESI for all samples were consistent with variations in the solubility curves. Near pH = 4, where the electrostatic repulsion between molecules is minimal, protein aggregation occurs, resulting in the lowest EAI and ESI values for all samples. Notably, FD-CPI had superior emulsification performance than SD-CPI and VD-CPI at all pH levels, which could be attributed to its higher hydrophobicity [45] and effective oil–water interface dissociation [46].

#### 3.2.3. Foaming Properties

The foaming properties of proteins depend on their adsorption capacity at the air–water interface, the ability to undergo rapid conformational changes and reorganization at the interface, and the capability to form viscoelastic films through intermolecular interactions [47]. As illustrated in Figure 6C,D, within the measured pH values, the trends in the FC and FS values of the samples generally mirrored the solubility curves. Minimal FC and FS values occurred near the pH values approaching the isoelectric point, primarily due to the lowest solubility, where high solubility in water is a crucial prerequisite for the development of high FC. Proteins with a higher solubility have interactions with water molecules, swiftly diffusing to the gas–liquid interface to form a protein-binding layer, consequently exhibiting superior FC and FS [48]. From pH 5 to 12, the FC and FS of all protein samples increased, possibly due to the structural expansion of proteins with increasing pH, leading to enhanced protein diffusion at the water/air interface [30]. Significant differences (*p* < 0.05) in FS were observed between VD-CPI and FD-CPI or SD-CPI. SD-CPI displayed the highest FC and FS due to its smallest particle size and having the highest solubility.

## 4. Conclusions

In summary, our results indicated that different drying methods (FD, VD, and SD) had significant impacts on the physicochemical and functional properties of CPI. Notably, compared to FD and SD, VD had greater effect on the color, surface hydrophobicity, intermolecular disulfide bonds, intrinsic fluorescence, and thermal stability of CPI. In addition, SEM results showed that the microstructure of SD-CPI was spherical, while those of FD-CPI and VD-CPI were lamellar and blocky, respectively. VD-CPI had the largest particle size (122.10 ± 4.33 nm), followed by FD-CPI (117.70 ± 1.66 nm) and SD-CPI (84.70 ± 1.22 nm). FTIR analyses revealed that VD-CPI had the highest β-sheet (44.47%) structure content but lowest α-helix (22.65%) and β-turn (13.53%) structure contents. Furthermore, the solubility, emulsification performance, and foaming properties of VD-CPI were inferior to those of FD-CPI and SD-CPI under different pH levels. For example, the solubility of CPI was 15.16%, 8.62%, and 6.10% higher after SD process in relation to the VD process at pH 6, 7, and 8, respectively. Consistently, SD-CPI exhibited a significantly higher emulsification performance and foaming properties than VD-CPI at these pH values. These findings hold significant reference value for the production and application of CPI. Specially, from an industrial-production point of view, SD-CPI is recommended for food applications due to its better functional properties and low production cost.

## Figures and Tables

**Table 1:** Protein content and color parameters of FD-CPI, VD-CPI, and SD-CPI.

Samples	Protein Content (%, Dry Weight Basis)	Color			
		L*	a*	b*	Δ*E*
FD-CPI	93.24 ± 0.30 ^b^	40.25 ± 0.13 ^b^	1.56 ± 0.49 ^b^	2.55 ± 0.06 ^b^	54.33 ± 0.11 ^b^
VD-CPI	87.52 ± 0.70 ^a^	35.81 ± 0.10 ^a^	1.65 ± 0.18 ^b^	4.05 ± 0.09 ^c^	58.80 ± 0.11 ^c^
SD-CPI	92.18 ± 1.46 ^b^	55.53 ± 0.49 ^c^	0.57 ± 0.12 ^a^	0.25 ± 0.06 ^a^	39.07 ± 0.48 ^a^

Means with different superscript letters (a, b, c) within the same column are significantly different (*p* < 0.05).

**Table 2:** -SH content, H_0_, particle sizes, zeta potential, Td and ∆H of FD-CPI, VD-CPI, and SD-CPI.

Samples	FD-CPI	VD-CPI	SD-CPI
-SH (µmol/g)	5.32 ± 0.01 ^b^	4.86 ± 0.03 ^a^	5.24 ± 0.11 ^b^
H_0_	85.99 ± 6.79 ^c^	52.59± 0.01 ^a^	70.60 ± 7.10 ^b^
Particle sizes (nm)	117.70 ± 1.66 ^b^	122.10 ± 4.33 ^b^	84.70 ± 1.22 ^a^
T_d_ (°C)	128.20	138.17	142.36
∆H (J/g)	98.79	117.43	101.38

Means with different superscript letters (a, b, c) within the same column are significantly different (*p* < 0.05).

**Table 3:** The secondary structure contents of FD-CPI, VD-CPI, and SD-CPI, determined via FTIR.

Samples	β-Sheet (%)	β-Turn (%)	α-Helix (%)	Random Coil (%)
FD-CPI	42.38	16.20	26.04	15.38
VD-CPI	44.47	13.53	22.65	19.35
SD-CPI	33.33	17.82	27.85	21.01

## Data Availability

The original contributions presented in the study are included in the article, further inquiries can be directed to the corresponding authors.
